# “It Is Difficult to Always Be an Antagonist”: Ethical, Professional, and Moral Dilemmas as Potentially Psychologically Traumatic Events among Nurses in Canada

**DOI:** 10.3390/ijerph19031454

**Published:** 2022-01-27

**Authors:** Rosemary Ricciardelli, Matthew S. Johnston, Brittany Bennett, Andrea M. Stelnicki, R. Nicholas Carleton

**Affiliations:** 1 Department of Sociology, Memorial University of Newfoundland, St. John’s, NL A1C 5S7, Canada; matthew.johnston3@carleton.ca (M.S.J.); bapbennett@mun.ca (B.B.); 2 Department of Psychology, University of Regina, Regina, SK S4S 0A2, Canada; andrea.stelnicki@uregina.ca (A.M.S.); nick.carleton@uregina.ca (R.N.C.)

**Keywords:** occupational mental health, potentially psychologically traumatic events, nurses, medical power, moral injury

## Abstract

Aims: We explore social and relational dynamics tied to an unexplored potentially psychologically traumatic event (PPTE) that can impact nurses’ well-being and sense of their occupational responsibilities: namely, the moral, ethical, or professional dilemmas encountered in their occupational work. Design: We used a semi-constructed grounded theory approach to reveal prevalent emergent themes from the qualitative, open-ended component of our survey response data as part of a larger mixed-methods study. Methods: We administered a national Canadian survey on nurses’ experiences of occupational stressors and their health and well-being between May and September 2019. In the current study, we analyzed data from four open text fields in the PPTE section of the survey. Results: In total, at least 109 participants noted that their most impactful PPTE exposure was a moral, professional, and/or ethical dilemma. These participants volunteered the theme as a spontaneous addition to the list of possible PPTE exposures. Conclusions: Emergent theme analytic results suggest that physicians, other nurses, staff, and/or the decision-making power of patients’ families can reduce or eliminate a nurse’s perception of their agency, which directly and negatively impacts their well-being and may cause them to experience moral injury. Nurses also report struggling when left to operationalize patient care instructions with which they disagree. Impact: Nurses are exposed to PPTEs at work, but little is known about factors that can aggravate PPTE exposure in the field, impact the mental wellness of nurses, and even shape patient care. We discuss the implications of PPTE involving moral, professional, and ethical dilemmas (i.e., potentially morally injurious events), and provide recommendations for nursing policy and practice.

## 1. Introduction

In Canada, there are a limited number of empirical studies documenting Canadian nurses’ possible struggles with mental health and potentially causal factors, such as potentially psychologically traumatic event (PPTE) exposure. Internationally, nurses have been found to have experienced symptoms consistent with a range of mental disorders [1,2,3,4]; symptoms that may develop because of, or at least in part to, their occupational work and environment [3,5,6]. In a recent Canadian study, researchers found that nearly half (47.9%) of the nurses in their sample of 4267 respondents screened positive for a mental disorder [7], with many experiencing difficulties with burnout [8] and suicidal behaviours [9]. Recognizing that nurses are exposed to PPTEs in due course by performing their occupational responsibilities, there is a need to better understand the social and relational dynamics in their work environment that may trigger or aggravate such exposures.

In Manitoba, Canada, research found that the five most impactful PPTEs or stressors were: (1) a child’s death; (2) workplace violence; (3) providing care for patients who resemble friends or family; (4) efforts in the preservation of life being unfruitful (e.g., patient dies or experiences reduced quality of life); (5) responsibility for too many patients [10,11]. Other identified PPTE exposures in this research included emotional or psychological abuse, physical violence, professional or personal conflict, and patient death [12]. The Manitoba Nurses Union [10] identifies that the omnipresence of potential violence is a risk factor for PTSD in their sample of nurses (e.g., 52% of respondents reported experiencing physical violence and 76% reported verbal violence). Emergency room nurses have sometimes been found to experience more aggression from patients than nurses in other departments (e.g., internal medicine, surgery, pediatrics, obstetrics/gynecology [13]). Other researchers also found that nurses were exposed to an average of 13.18 (*SD* = 4.35) of the 20 diverse PPTE types [14]. Moreover, “across the entire sample, the PPTEs most frequently identified as the worse event were physical assault (12.2%), followed by the death of a child (10.9%), and death of an individual after extraordinary efforts were made to save their life (10.8%)” [14] (p. 4).

Departing from this body of work that identifies how some nurses can experience compromised mental health when exposed to PPTEs at work, we explore the less known ways nurses experience moral injury and distress as potentially psychologically traumatic. We suggest that physicians, other nurses, staff, and/or the decision-making power of patients’ families can negatively reshape nurses’ perception of their agency, which directly and negatively impacts their well-being. We structure the current article into four parts. First, we present a brief literature review framing what is known about the experience of moral injury among nurses, as well as the social relations among medical providers and the realities of the interactions between patients’ families and nurses. Second, we present our methods, before introducing our findings. We then conclude the paper by discussing some cautious recommendations that first acknowledge moral, ethical, or professional dilemmas as PPTEs and, second, propose solutions that address such PPTE exposures among nurses, both in terms of their mental health and professional needs.

## 2. Background

### Social Relations of Nursing: Hierarchy, Interaction, Moral Injury

By virtue of their medical knowledge and training, physicians hold legitimate and expert power over their patients and, in some cases, over other professionals. As such, physicians may intentionally or incidentally suppress their patients’ decision-making capacity or subordinate other healthcare workers, such as nurses [15]. Nurses work under the control, governance, and supervision of physicians (and other nurses) and routinely observe (usually more than physicians) their patients. With the assistance of other allied health professionals and care workers, nurses are trained to engage in a practical, hands-on, patient-centered, round-the-clock module of care that eventually builds in them their own medical expertise. Alongside their professional knowledge, nurses are still generally expected to overtly defer to the orders and recommendations of physicians under strict and established divisions of labor [16,17,18,19].

Some research shows that recalcitrance against established medical hierarchies could jeopardize a nurses’ career, especially if the nurse lacked social capital or such insubordination was not socially enacted in an appropriate, subtle, selective, informal, and/or sensitive manner [15,20,21] (The *Code of Ethics for Registered Nurses* from the Canadian Nurses Association states, “If a nurse encounters a situation where harm is underway or there is a clear risk of imminent harm, the nurse takes immediate steps to protect the safety and dignity of the persons receiving care. Some examples of appropriate immediate steps in cases of actual or imminent harm could include, but are not limited to, speaking up if a potential error in drug calculations is detected, questioning an unclear order, intervening to prevent unsafe restraint practices, protecting patients when a colleague’s performance appears to be impaired for any reason or intervening in a serious breach of confidentiality involving people with sexually transmitted infections” [22] (p. 33). Informally in hospital spaces, when a nurse disagrees with the doctor’s order and the issue cannot be immediately resolved, they may take the problem to their nursing manager, supervisor, or Director of Care. If neither of those parties can resolve the issue with the physician, they can contact the Medical Director). One key ethnographic study of Ontario-based registered nurses, registered practical nurses, personal support workers, and other healthcare workers demonstrates the gendered and increasingly racialized ways care work can materialize in long-term care facilities and how this problematic trend can impact workers’ experiences of health and safety [19]. More specifically, some nurses in their study reported experiences with short staffing, high workloads, bullying from superiors, and moral suffering over the ways colleagues were perceived to be unappreciated and underpaid. Similarly, other scholars found that long-term residential nursing staff in four Canadian provinces experienced declines in their psychological health and well-being when they experienced work role overload, low worker control, disrespect, and discrimination [23]. What exacerbated these unfortunate realities for many nursing staff was that many nurses perceived that they were largely on their own to cope with difficult workplace conditions, and that their employers and even nurses themselves often mis-recognized or ignored this phenomenon.

The hierarchical context under which nurse–physician relationships are formed can also impede nurses’ professional discretion [24]. The professional discretion includes autonomy to improvise, make assessments, and engage in conduct based on personal intuition, judgment, and experience rather than organizationally defined regulations [25]. The presence of autonomy and discretion are usually associated with a healthy working environment [26,27]. Many studies indicate that the work of nurses often involves a complex interplay between practice, culture, and accountability [28,29,30,31], meaning that on-going power struggles between nurses, physicians, patients, and/or family members vastly shape nursing practices and patient care. Nurses recognize that care work demands the use of professional discretion to prevent hospitals or other healthcare spaces from becoming spaces of indifference [29] or mundane, task-oriented, more detached systems of care delivery [32]. Nurses are simultaneously highly accountable healthcare agents who can be held liable by, or be reprimanded by, physicians, family members, and the public for medical errors, lapses in judgement, or professional slippages [16].

Some nurses—especially early in their careers—may be less confident about asserting themselves in discussions about patient care with physicians [33,34,35]. When nurse–physician relationships are not centered around trust, open communication practices, and transparency, or evolve into competitive struggles, patients often experience negative outcomes for care and confidence in medicine, and nurse retention suffers [36,37,38,39,40]. Collaborative input from nurses at every stage of patient treatment could improve care and make healthcare spaces a more creative and less dysfunctional place where nurses and physicians openly share and critically reflect on their clinical opinions and activities [24]. Fewer hierarchal communication styles can significantly enhance nurses’ job satisfaction and perception of nurse–physician relations [41]. Many healthcare facilities have policies for written communication between nurses and physicians, but the more common verbal communication between nurses and physicians in times of urgency and uncertainty is less regulated and potentiates negative patient outcomes and communication breakdowns, especially when the healthcare facility is busy, overcrowded, and chaotic [42].

The involvement of families in the treatment of their loved ones also shapes the social dynamics of care. One study of Canadian psychiatric nurses found that family members’ sense of entitlement, confrontational behaviour, and many demands contribute to nurse frustration and fatigue [43]. The same results identified a disparity between family expectations and the medical realities surrounding the patient’s circumstances, needs, and prognosis. When nurses tried to work through these differences in perception, family members were reported to sometimes belittle nurses’ judgment of the situation and called on physicians to support what family members believed was the proper course of action for the patient. The participating psychiatric nurses also remarked on how patients’ families tend to project and misplace their emotions onto nurses [43,44]. Relational tensions, alongside problems related to a lack of medical and healthcare resources available to nurses, can exacerbate strained communication practices between nurses and family members [45].

Nurses may also perceive patients’ family members as requiring care [46]. Nurses can provide imperative emotional support for families in times of need, use their intuition to assess patient needs, provide patient care and assurance, as well as work to understand their positions on the patient’s treatment plan [47]. Nurses also have flexibility in allowing patients’ family members to be present during resuscitation efforts, which is a common request [48]. Many nurses acquire additional education through workshops, conferences, and training programs to envision a preferred future based on what matters to families in approaches to care [49].

Nevertheless, nurses and families still experience an emotional toll and psychological distress when family members’ expectations and needs are unmet, and when nurses’ professional knowledge, skills, and judgments are not recognized or respected by physicians or family members, sometimes at the expense of patient care and safety [50]. Such unfortunate predicaments or tensions can make nurses feel as though there has been a betrayal of what is right by people in a position of legitimate authority over them, or by one’s self, also known as “moral injury” [51]. Morally injurious events refer to experiences wherein a person does, or fails to do, something that violates their morals, ethics, or personal values, and can produce symptoms consistent with one or more different mental health challenges (e.g., burnout, posttraumatic stress disorder) [51,52]. Potentially morally injurious experiences among nurses can result in moral injury symptoms including guilt, shame, spiritual–existential crisis, loss of trust, depression, anxiety, anger, self-harm, and social problems [51,52]. As dedicated healthcare professionals often navigating shifting protocols, shortages of resources, and high numbers of patients requiring care during time constraints, nurses experiencing moral injury can simultaneously feel a sense of powerlessness when they believe they are unable to provide the best care for their patients [53].

## 3. The Study

### 3.1. Aims

We administered a national Canadian survey on nurses’ experiences of occupational stress and their health and well-being between May and September 2019. The current study explores how interactions among, and directives from, physicians and patients’ families are currently being negotiated, problematized, and adhered to by nurses in Canada, which often places nurses in a position of moral, ethical, or professional conflict and injury. Unfortunately, relational tensions often impact the patient’s well-being as well as that of nurses—our focus here is on the experiences of nurses. In the current study, we examine what constitutes nurses’ exposure to the PPTEs that they identify as the moral, ethical, and/or professional dilemma (i.e., a PPTE that is also a potentially morally injurious event) to discuss policy and practice changes that could address the resultant implications. We look at how physicians, nurses, staff, and/or the decision-making power of patients’ families can inform these dilemmas as nurses are often feeling left without agency, operationalizing care instructions with which they disagree, and, as a consequence, can experience these events as PPTEs. We also look at accidents and errors in judgment that create such dilemmas [14].

### 3.2. Design

We used a semi-grounded theory approach [54,55] to analyze qualitative responses from the open-ended component of survey items that followed the presentation of the Life Events Checklist (LEC-5), which asked about exposures to PPTEs [56]. We also used the PTSD Checklist for DSM-5 (PCL-5) to assess PTSD symptom severity over the last month to the LEC-5 event participants found most stressful or was causing the most interference.

### 3.3. Sample

There were 7358 participants (92.0% women) who started the survey and 4067 (94.8% women) completed the relevant measures on PPTE exposure section, where participants were offered the option of providing additional information on their PPTE exposures through a qualitative, open text response field. In 2019, 91% of regulated nurses in Canada were women. Though nursing continues to be a female-dominated profession, the supply of male regulated nurses increased by 15.4% between 2015 to 2019 [57]. In the current study, we used data from one coded theme of “ethical and moral dilemmas”, which consisted of 120 participant responses (a total sample of 109 participants, since 11 responses were from the same participants in different open text fields). Please see Table 1. for the participant characteristics.

### 3.4. Data Collection

We obtained our data through a web-based, self-report survey that was administered through Qualtrics to Canadian nurses between May and September of 2019. We offered the survey in both French and English using validated measures paralleled from a previous survey of Canadian public safety personnel [58]. We revised the survey in collaboration with the Canadian Federation of Nurses Unions’ (CFNU) executive team to ensure the design included relevant nursing variables. The CFNU distributed the survey to their member unions’ email accounts and advertised the survey on social media pages, including a video by the CFNU President that encouraged nurse participation. Participants accessed the study through the provided website link where we issued a unique computer-generated random code to facilitate anonymous, repeated entry into the survey. Participants provided informed consent before entering the survey.

Lifetime exposures to PPTEs were measured with the Life Events Checklist 6 (LEC-5) [56]. See Stelnicki and colleagues [14] for details on how the LEC-5 was used and augmented within the current survey. Within the PPTE exposure section of the survey, there were five open text fields where the participant had the option of giving additional details, four of which were used within the current analysis. The general PPTE exposure section open field “PCL5 Comments” was not used for the current analyses because the field focused on “stressful events” rather than PPTEs.

The LEC-5 list that was used consisted of 17 questions with four nurse-specific additions. The participants were asked to indicate that each event happened in one of six ways: “happened to me”; “witnessed it”; “learned about it”; “part of my job as a nurse”; “not sure”; “does not apply”. The last event listed was “any other very stressful event or experience” (i.e., LEC-5 item 17) and participants were asked to provide details in an open text field. We included data from the details provided in LEC-5 item 17 that were identified as a part of their job as a nurse in our analysis. The following component of the survey asked participants to choose the event that was the worst, the most distressing, or causing them the most difficulty currently. At the end of this section, participants were asked to provide additional comments regarding their exposure to potentially psychologically traumatic events (LEC-5 item 4). We also included data from LEC-5 item 4 that were discussed as a part of their job as a nurse in our analysis.

Within the PPTE exposure section, participants were asked about the frequency and difficulty of events specific to their nursing career. Fourteen questions asked about nursing-specific PPTEs in addition to an “other” open text field (i.e., “NursingExp Other”) where the participant was asked to explain. We included data from the NursingExp Other section in our analysis. At the end of the PPTE exposure in the nursing career section, participants were asked “do you have any other comments regarding these events?” (i.e., “NursingExp Other”). We also included data from the “NursingExp Comment” open text field in our analysis.

### 3.5. Ethical Considerations

Ethical committee approval was granted by the University of Regina (File #2019-052) as well as CFNU. To support pan-Canadian participation, we also reached out to non-CFNU member nurses’ unions in British Columbia and Quebec, as well as a limited amount of nursing associations and colleges.

### 3.6. Data Analysis

We analysed all open text field responses in the survey using a multi-phase, semi-grounded constructed approach for the larger study [54,55]. We applied open coding to all qualitative questions that were a part of the survey to classify the participant responses into emergent themes using the coding software program NVivo (available through QSR International at https://www.qsrinternational.com/, Doncaster, Australia). Each survey question was organized by survey section to prioritize analysis for publications (i.e., we used data for the current analysis from questions under the PPTE Exposure section of the survey). Within this coding process, parent codes were created and reorganized as sub-themes that emerged from within the data until a saturation of themes occurred. We first pursued initial coding that focused mainly on organizing the parent codes of the data. We then went back through the parent codes through a process of secondary coding to break down those major themes into more clear and concise sub-codes. During the secondary coding process, codes that were similar or overlapping were merged, and sub-codes that were not frequent or meaningful were dropped. The coding was based on four out of five open text questions in the PPTE exposure section of the survey (i.e., “LEC-5 item 17”, “LEC-5 item 4”, “NursingExp Other”, and “NursingExp Comment”).

In the second phase of our analysis, we merged the coded themes of LEC-5 items 4 and 17; in addition, we merged NursingExp Other with NursingExp Comment. We then extracted the work-related responses from LEC-5 items 4 and 17, and merged the coded themes with all the coded themes in NursingExp Other and NursingExp Comment, creating a nine-code scheme based on the most emergent work-related PPTE exposure discussed in these four open text fields of the survey. We extracted the codes under one of the nine parent codes labelled, “patient care” and focused solely on the emergent sub-code “ethical and moral dilemmas”. These codes were exported out of the NVivo software and manually reorganized to analyze the different ways nurses experience ethical, professional, and moral dilemmas as PPTEs. In total, 120 participant responses were coded to the theme in the question asked. Nurses also mentioned the same theme in responses to other questions (thus, in a different context), which were excluded from the current analyses. Nurses were never asked in the survey about moral, ethical, or professional dilemmas; accordingly, the moral, ethical or professional theme across respondents was emergent and often overlapped with reports of professional internal conflict.

### 3.7. Validity and Reliability

The nurses who provided qualitative data (*n* = 4067) also reported being exposed to multiple and varying PPTE categories, often 11 times or more. There were statistically significant (*p* < 0.05) associations between diverse PPTEs and positive screenings for all mental disorders (i.e., posttraumatic stress disorder, major depressive disorder, general anxiety disorder, panic disorder) except alcohol use disorders [14]. In the context of such results, we assessed an emergent theme that was not captured in the quantitative data; specifically, the moral, professional, and/or ethical dilemmas that many nurses volunteered as being their most problematic impactful experience. Due to the nature of analyzing the additional comment portions of the survey, participants volunteered the information, having it come to mind outside of the list of the possible PPTE exposure they routinely endure.

## 4. Results

The participants included in the current study were largely female (97.5%) across varying age groups, the most were between 30–39 (24.2%). Many participants reported working for 10 or more years (54.1%), and most reported working as Registered Nurses (90%).

### 4.1. Ethical, Professional, and Moral Dilemmas

In summary, nurses replied candidly that their PPTEs resulted from managing ethical, professional, and moral challenges or “ethical dilemmas” (P2277), which refers to the psychological impact of having to act different from what one feels is morally, ethically, or professionally appropriate. Our findings reveal that nurses who disagree with a course of action prescribed by a physician, requested by family members, or forced by resource limitations, become professionally, ethically, and morally conflicted or injured. The conflict is based on nurses not feeling able to provide adequate or necessary patient care in ways that align with their professional and moral standards. Nurses’ reported experiences that are complicated and informed by diverse beliefs tied to religion, values, education, and many other shaping factors [59] that are also important to analyse sociologically but beyond the scope of the current study. Nurses in our study described diverse ways of experiencing their most distressing PPTE, which was often linked to providing patient care with little room for agency that draws on their extensive professional knowledge and experience.

In the sections that follow, we first unpack the PPTEs nurses experience that result from their lack of agency to enact or challenge the treatment plans of physicians, particularly in situations where the professional knowledge or assessment of the nurse is disregarded by physicians who serve in positions of high medical authority over nurses. We then turn to the ethical, professional, and moral dilemmas that emerge from inadequate human or material resources, specifically when medical staff are obligated to choose who (and in what order) patients receive sometimes life-saving care. Finally, we address the PPTEs underpinning nurse–family relations as family members struggle with, or fight to, prolong the life of a terminal patient; what nurses consider to be professional errors or misjudgment; when a seemingly coherent patient loses their autonomy to make end of care-informing decisions (e.g., the power of attorney denies the patient’s wishes and redirects the course of action).

### 4.2. Disregarded Assessments and Lack of Agency in the Context of Medical Power

In following the instructions of care as prescribed by the treating physician, nurses described how they worked through moral and ethical challenges that they often experienced as PPTEs. A nurse points towards the PPTE that resulted from what she perceived to be a physician’s failure to provide care:

*The patient had surgery to remove gall stones and returned. Overnight the patient’s developed requirements increased and troponin was elevated. Doctor was made aware three times overnight and did nothing. Said it was sepsis. By 10am the following morning the patient died (P2220).*

Participant 2220, echoing many other respondents, felt conflicted because an attending physician failed to respond to their assessments and recorded changes in the well-being and status of the patient. In each situation, the nurse felt their interpretation was regarded by the physician as insignificant and, as a result, the patient suffered and may have died unnecessarily. The fact this nurse (and possibly others) made the physician aware of the patient’s changes “three times” demonstrates the willingness of the nurse to advocate for their patient and redirect physicians’ potential professional miscalculations, but, in this case, such persistence was overshadowed by the power of the physician to ignore or not confirm the nurse’s recommendations. Following such events, nurses are often confronted with the difficult professional burden of explaining to the patient’s family or loved ones the incident and reasons surrounding the physician’s decision. The dilemmas regarding such explanations are also PPTEs that can place nurses, physicians, and senior healthcare officials in a position of liability if they do not openly justify and support the physician’s prescribed course of action. Describing how physicians can ignore the nurses’ assessments, other respondents explain:

*Doctors not responding to assessments and patients dying because of it (P2973).*

*Part of a breach birth that had a negative outcome that was avoidable if the physician would have listened to nursing concerns. It makes it very difficult to work with that physician. It causes flashbacks of the incident and anxiety in myself (P3011).*

*There have been decisions that physicians have made that have not been supported by nurses (i.e., changing meds not being in the best interest of the patient). I’ve had a patient who was very quick to deteriorate with med adjustments… It was difficult to not feel like we had created that situation and indirectly caused the outcome for someone who had been doing well, and was only following doctors recommendations (P1203).*

In the first excerpt, Participant 2973 bluntly described the direst of consequences when care was inadequate—the patient died because her assessments were not given appropriate consideration by the attending physician. In the second excerpt, Participant 3011 describes the PPTE that resulted from a physician failing to treat her concerns as significant. Participant 1203 described how a physician not attending to the nurse’s concerns created a more adverse situation for the patient. The theme across the excerpts is how physicians not attending to the nurses’ concerns can result in the deterioration of patient conditions.

Participants 3011 and 1203 both describe tensions that erupt from “following recommendations” that harmed patients. Participant 1203 responsibilizes herself “indirectly” for not “doing more” in what is framed by Participant 3011 as a “difficult” and seemingly impenetrable medical hierarchy and culture of decision making, entangling relations between nurses and physicians. In all of the passages quoted so far, nurses were not silent about their disagreements with physicians even though such dissent was ignored, but despite their repeated assertions, their sense of agency becomes lost as they are left to ultimately fulfill the competing prescriptions of physicians, sometimes at the expense of the patient.

Other nurses discussed how situations unfolded when the physician fought to preserve life in circumstances where the patient’s death, from the perspective of the nurse, was inevitable. For example, Participant 1798 described the process of admitting a patient with “extensive traumatic brain injuries and physical injuries”, all of which had quickly been deemed “non-survivable by neurosurgery and our intensivist fellow”. She continued to explain that the physician assigned to the patient “wanted to do everything we could to keep the patient alive”. The nurse depicted the event as a PPTE:

*I was so busy tending to the patient and completing physician orders and barely meeting strict goals that in a 12 h shift I couldn’t take a mere 10 min to just be with the obviously devastated family, who were sobbing at his bedside. Instead of letting this man die peacefully surrounded by his family we took him away to IR, CT, etc., leaving fewer moments for them to be with the patient. Looking back, I’m angry and saddened about how aggressive our care was for a man that could not be saved. It robbed him of his dignity and his family’s ability to mourn his approaching death. The patient passed 2 h after I reported off to the day nurse.*

Participant 1798 describes the personal impact of having to obey the instructions of the physician, and the moral and ethical dilemma that arose as she did her job feeling as though she was pointlessly and “aggressively” stealing precious final moments away from the family and violating the patient’s “dignity”. In this case, physicians themselves disagreed about the treatment course, and despite the fact that neurosurgery deemed the case as non-survivable, the attending physician was still empowered to try to preserve their life for as long as possible. The nurse was caught in the middle, and became routinized with physicians’ orders and directions, unable to console the nearby family members who seemed to bear little impact and influence on the decisions made by medical personnel. The nurse expressed anguish at having to carry out their medical responsibilities instead of attending to the interpersonal, social, and relational dynamics that accompany nursing work and patient care.

Other examples in the data include Participant 2001 who reported that, “There are a couple of our physicians who refuse to let patients go. They treat and treat and treat to the point of patients who should be palliative in our care.” Such practices extended beyond acute care situations to long-term and palliative care provision, where nurses believed their actions were causing patients undue suffering at times. The challenge was complicated by nurses being professionally obligated to adhere to the physician’s instructions despite their differing personal interpretations of the situation:

*It is difficult to watch how we currently extend the life of our palliative patients through medical intervention. I see daily a lot of prolonged suffering where nurses are often questioning the orders as we know there is no hope (P2919).*

*Being forced to give prescribed dose of medication knowing it will kill your terminally ill patient (P2373).*

Participant 2919 describes the prolonged suffering endured by some in palliative care as a PPTE. Physicians should try their best to save a patient’s life, but as caregivers who attend to their patients round-the-clock and see more visibly and consistently patient suffering, nurses may conceptualize death as sometimes being better than “a lot of prolonged suffering”, which can conflict with the attitudes of many physicians.

Participant 2373 described ending the life of a patient who was terminal. The obligation of giving the fatal dose of medication that the physician prescribes to the patient does not sit well with this respondent. As evidenced by “forced”, the nurse was not provided with the opportunity to meaningfully discuss her role and professional opinion in this course of action, and instead had to preserve medical authority and become, in their words, a medical instrument in the “killing” of a patient. In both cases, the nurses lacked agency, control, and authority because their occupational responsibilities obligated their actions, which the nurses framed as morally injurious.

### 4.3. Witnessing, but Rarely Curbing, Physicians’ Behaviours

Nurses also described witnessing other select behaviours of physicians as PPTE. One nurse wrote of “*watching physicians fail to fully inform patients of high risk in elective procedures, then have them die on table” (P2257)*, or make promises that they could not keep and should never have made in the first place. For example, Participant 830 described a PPTE that occurred when a male patient presented with cardiac arrest. She *“witnessed [the] Cardiac interventionist tell his family he would be ‘fine’ as they signed consent for cardiac stent. I helped wheel him to cath lab...where he died. I still feel so bad about that, I’ll never forget the way he told them their loved one would be ‘fine’”.* Participant 830 continues to live with the aftermath of her experience, one in which she had no control or authority to intervene or rectify. Here, a few casual but significant words about the nature of a risky procedure evolved into a professional slip and miscalculation that cost the patient their life. Again, the nurse expresses regret, guilt, and distress over being forced to take a role in this event where they had to “wheel” a person to their place of death.

Some nurses also reported about physicians employing “fear tactics”, which compromised the process of acquiring informed consent:

*Client’s right to informed consent is not always respected, and fear tactics are sometimes used. This is really upsetting to me, but it is difficult to always be an antagonist with people I have to have a working relationship with (P2835).*

In the excerpt above, the nurse describes how some physicians, who in trying to preserve the best care and interests of their patient, may simultaneously compromise the patient’s decision-making capacity through pressure or “fear tactics”, especially if the patient is unwilling to undergo such treatment. This nurse is left morally, ethically, and professionally compromised, unable to intervene in the authority of the healthcare provider but still uncertain about their own positionality and the actions they watch their patient agree to in “fear”. Unlike other nurses in this study, this respondent constructs their internal disagreement as potentially “antagonizing” rather than virtuous, and worries that constant resistance may jeopardize an already tricky and tenuous relationship with the physician who they must still rely on to direct their actions.

Nurses reported experiencing PPTEs while working in childbirth and delivery; specifically, they witnessed behaviours by physicians that compromised the dignity, safety, consent, and discernment of the patient. One nurse described the vulnerable state of a mother and infant, underscoring that women giving birth for the first time in particular may not realize that the actions of the physicians are in violation of acceptable standards and practices:

*Emergency vaginal/Forcep deliveries for women to deliver their babies alive when pain medication is not able to be given timely/pain medication isn’t effective at all. Feels like an assault when the women are screaming and yelling. I’ve also seen doctors pulling on and stretching women’s perineum during pushing—assault and women who are having their first baby think it’s normal. Doctors/residents who will do a cervical exam by putting their whole hand inside the woman’s vagina—should never be more than 2 fingers unless the woman is bleeding to death and needs a manual removal of her placenta (consented to by patient) (P2319).*

This nurse perceives the execution of an invasive medical procedure as commonly crossing professionally established boundaries to the point that they constitute an “assault” on vulnerable women who may not know what acceptable and unacceptable medical practice is. In this case, “screaming and yelling” does not indicate strongly enough to the attending physicians or residents that what they are doing is violating the safety and dignity of women who rely on, and likely trust doctors to perform only what actions are necessary, with the patient’s consent, to facilitate a safe birth. Knowing the acceptable procedures and specific reasons that would justify a more invasive procedure, the nurse expressed serious discomfort with the “normality” the patients often ascribe to the indefensible actions of physicians, despite there being patient experiences of serious pain and distress. Relatedly, patients undergoing treatment in other domains of medicine (e.g., psychiatry) have told healthcare providers that receiving chemical injections of sedatives in their exposed buttocks for disruptive behaviour feels like a sexual assault [60]. This excerpt exposes the sharp interpretive skills of nurses to distinguish between justifiable and unjustifiable medical practice, but who, unfortunately, have little recourse to redirect, intervene, or condemn the actions of physicians without jeopardizing their job security or working relationships.

### 4.4. Inadequate Resources and Life Changing Decision Making

Nurses expressed their exposure to PPTEs when being placed in situations where they have to determine who receives care and in what order, knowing that greater injury or death can be experienced by those waiting for care. For example, Participant 1012 wrote: “Choosing who gets care and who waits knowing some that wait may die.” Nurses explained that care is sometimes limited by staffing and capacity challenges. The limits can create a prioritizing of patients based on diverse criteria (e.g., age) that can compromise care and the professional standards of nurses. Such experiences were felt across different branches of hospitals and healthcare institutions, impacting nurses across the country:

*Working in a specialized geriatric unit that is constantly understaffed and over capacity I see situations that are stressful every day where patients are not taken care of in a priority manner and they end up passing away due to lack of priority due to their age (P3908).*

*Feeling like you can’t provide quality care in hospital due to circumstances beyond your control (P3097).*

*Death of patients due to overcrowding, cuts, lack of adequate equipment or staff (P2249).*

In the first excerpt, Participant 3908 describes the age hierarchy that sometimes determines care allocation. Participant 3097 and Participant 2249 describe how human or material resource shortages can determine care and influence mortality. Accordingly, nurses struggle with PPTE and the moral implications of deciding who lives and who dies because of resources.

Participant 2596 writes of the PPTE that comes from “upholding unpopular and inequitable health care services and circumstances”, describing the experiences as the most stressful PPTE of their career. In all of these quotations, nurses identify the paradoxical power shift that can occur when responsibility for difficult decisions is left with the nurses instead of the people more responsible for making such decisions. Participant 2596 alludes to the decisions being shaped by bureaucratic policies from healthcare administrators that cause nurses to feel responsible for making inescapable and “inequitable” decisions over which they have no control. Here, the challenge is having to deal with the responsibility allocated to them to determine who does and does not receive care.

### 4.5. Enacting the Wishes of Family to Prolong Life

An overwhelming number of nurses described their central PPTE exposure as resulting from the “moral distress” (P2258) and professional conflict they experience when asked by family members to prolong life in palliative situations:

*Moral distress—torturing a patient with “care” and procedures desired by the family that only cause harm and suffering. There is a desperation from the families to prolong an unprolongable life (P2258).*

*Torturing someone with invasive life support measures that only the family deem to be appropriate, despite a mountain of medical evidence to the contrary… We who treat suffering patients feel unable to speak up in advocacy of a patient whose family knows them best, and feels they know what their prior wishes would have been (P2317).*

Participant 2258 explains her PPTE that resulted from desperate families wanting to try anything to secure extra time with a fatally ill family member. Participant 2317 describes the PPTE involved with providing what the nurse believes will be futile, painful, and invasive treatments to a dying patient. Participant 2317 then describes the PPTE of being unable to advocate for patients. The participant reports a PPTE involving experiencing internal conflict while negotiating their more objective professional assessment against the important subjective positionalities of the families. Families personally know the patients (and their wishes) better than their nurses; nevertheless, participants conveyed how families became conflicted by their desire to keep their loved ones alive at all costs. All three participants indicated the families of patients, rather than the physicians, were creating the PPTE conditions that can cause the moral challenges—a process of “care” two nurses refer to here as “torture” because of suffering caused to the patients. Relatedly, the decisions made by patients’ families appear to create situations of conflict and potential psychological stress as the care provided moves away from being centred on the patient and instead caters to the family:

*Keeping a patient alive when the patient is incompatible with life only to discontinue care 70 some days later once family is on board (P2338).*

*So many times in my career I am forced to give care that is futile, painful and won’t save or give my patient a good quality of life … family prolonging dying man’s life through artificial means and being extremely controlling and unreasonable, they were there 24/7 for close to 2 years (P2643).*

*It was a patient who was nearing the end of her life and her family demanded and expected unreasonable efforts made. They were also rude & confrontational with Staff. I feel it was unethical what was being done (P3173).*

In the first excerpt, the nurse describes the PPTE involved in watching family grievance processes, including the PPTE described by Participant 2643 in giving “futile, painful” care to a patient. Participant 2643 then describes the challenges of performing life-prolonging interventions and the strain of needing to watch a grieving family who received unwarranted hope for two years. In the final excerpt, Participant 3173 describes being required to act counter to her professional judgement and families treating her poorly.

Across the excerpts, there is a complex interplay of nurses’ actions and family actions. As was the case for nurses’ relationships with physicians, there appears to be a struggle for power triggered by desperate family members becoming “unreasonable, rude, and confrontational” with nursing staff. Nurses again framed themselves as having to navigate diverse social pressures and medical responsibilities that can compromise patients and their families, thus increasing their suffering. Participant 2751 echoed the challenges by describing the difficulty nurses face in “advocating for [their] patient while still respect[ing] family centred care initiatives”. Balancing two seemingly contradictory models of care is difficult and can create a PPTE.

### 4.6. Professional Error

Nurses also described instances where they personally acted in error. Explaining the PPTE as the most challenging in their biography, nurses explained:

*Patient died from error after I referred…Totally preventable (P1169).*

*Patient(s) dies or had serious injuries due to (what I think) me not doing my job optimally (P2663).*

*I was working ICU, and gave the wrong blood to a patient. That patient almost died. Only time in my life I considered suicide (P1162).*

*Witnessing loss of life of patients that I felt could have been saved by medical intervention (P844).*

The four excerpts are related in that the nurses question their own actions. In the first, Participant 1169 describes how she encountered the PPTE of a patient dying because of her error, feeling the responsibility and associated burden on herself. Participant 2663 then describes the harm from “not doing my job optimally”, while Participant 1162 explains that she considered death by suicide after her professional error. In the final excerpt, the nurse expresses the PPTE from not acting.

Each nurse’s most distressing PPTE is tied to their own actions and what is considered by them to be a professional “failure”. Respondents expressed turmoil and anxiety over not having their voices and professional opinions legitimated by physicians and family members, and over the reality that they can also make critical mistakes. Processing such errors is a cumbersome struggle that can make nurses question their own competency and intrinsic value as a human being, which can create compounding stressors. Other nurses expressed that the errors of their colleagues were most distressing. Nurses described being “part of a code that went seriously wrong” (P1446), “procedure errors” (P1499), and:

*Responded to a trauma code the patient was unresponsive and posturing and remains with severe brain damage (May still be in a coma). There was only one other nurse who did questionable treatments and disagreed with me and took over moving the patient without following spinal precautions etc. (P2714).*

Participant 2714 expresses the PPTE involved with professional disagreement and the impact that disagreement had on the well-being of the patient. What is consistent is that the actions of their fellow nurses resulted in their PPTE (e.g., “Death of patients or serious harm caused to patients as a result of co-workers error” [P2026]). Up to this point, we have articulated how it is often medical personnel other than nurses or family members that create problematic working environments for nurses, but here, nurses sometimes must deal with a break in their own solidarity that can create confusion, chaos, and stress.

Nurses also reported more about the medical errors they witnessed at the hands of physicians as their most distressing PPTE. The actions, often described as “medical errors with bad outcomes” (P3608) affected respondents on many levels: Professionally, in terms of their interest in optimally caring for the patient; emotionally, given the effects on the patients; personally, given they felt for persons who trustingly underwent surgical procedures under the care of the specific physicians. Such experiences ranged from physicians’ prescription practices (e.g., “Patient died after copious medications changes by different physicians to the point of neglect” [P1152]) to “surgical negligence, abuse of on call staff …acceptance of planned high risk surgical cases when theatres and staff are not equipped to do so, anesthesiologists who leave the OR during surgery, anesthesiologists who under sedate patients thus putting everyone at risk during the surgery…” (P4025). Another nurse explains her own PPTE from these types of incidents in more depth:

*A surgeon that caused injury and death for years at our hospital…It was a horrible time in my career. Very hard to care for his post surgical patients and watch them suffer. Also very hard to not warn the innocent public but if you did your entire career was in jeopardy (P2534).*

Nurses also implicitly described challenges with physicians’ orders and decisions that can result in professional repercussions for the nurses. The nurse explicitly indicates that coming forward about frequent medical neglect in the health care system could jeopardize their own career. Despite potential consequences, nurses reported wanting to educate the “innocent” public about possible risks.

Many participating nurses’ words in this study seem to imply that they are at the “brink” of resisting poor medical practice more overtly to preserve patients’ lives or dignity. One nurse described their emotions and actions after witnessing perceived malpractice:

*The patient had been in unimaginable pain for HOURS and had deteriorating vital signs for a long time until the doctor on the unit actually decided to transfer her. The patient’s family were traumatized from the event and upset nothing had been investigated earlier. This was extremely upsetting to see as my patient suffered a great injury, and my co-worker let her down. It made me feel guilty for weeks as I wondered if there was anything I could have done differently. I visited her in CCU frequently and had insomnia for many nights due to this patient harm (P2026).*

This nurse had to manage the emotions of the suffering family as well as their own feelings about the provided care. Unlike nurses, physicians appear to be heavily shielded from criticism, even when families are pushing for an “investigation”. The nurse appeared to problematically take on and process the PPTE and associated guilt, instead of that responsibility being taken on by the physician. The nurse showcases how the emotional spillage and wreckage resulting from poor medical practice can trickle down through nurses, creating additional PPTEs and burdens that can cause psychological harm.

### 4.7. Not Adhering to Patient’s Wishes

Many nurses described events where they were not adhering to patient wishes as the most distressing PPTEs. During such events the nurse can be caught between the authorities held by the family, the physician, and the patient, and still be forced to act against the patient. Situations involving end of life care or children appeared particularly stressful. Participant 1346 describes a patient who, despite the self-made DNR, was ventilated to prolong life:

*Pt had made self DNR but family rescinded it when patient not capable anymore, so they got attached and lived on a ventilator for 9 months begging to be allowed to die. Family never visited.*

In cases such as the one described by Participant 1346, nurses provide life-prolonging interventions that cause patients to suffer and nurses to struggle with directly conflicting patient requests:

*Watching my patients suffer because their family is not realistic about goals of care. Continuing full medical interventions when patients tell me they don’t want to or ask me to stop but unable because the family or decision maker has decided to continue (P2262).*

*The person involved was deemed unable to make decisions for their own health care, but was alert and vocal (P1827).*

*Very difficult to lose any patient, it is emotionally and ethically challenging when family change patient’s wishes of DNR to full code post patient losing consciousness (P3842).*

Participant 2262 describes not being able to act according to the patient’s wishes because the family has power of attorney, a situation similar to that of Participant 1827 who described a patient who was “alert and vocal”, but could not control their treatment plan. Participant 3842 described emotional, ethical, moral, and professional strains of not adhering to a patient’s wishes because of a decision made by family. The common narrative is that nurses experience a PPTE because they are unable to follow the wishes of patients due to mandates over which the nurses have little agency to negotiate.

## 5. Discussion

The literature on PPTE exposure among first responders, other public safety personnel, and care workers is growing to better understand how PPTE exposure impacts the psychological well-being of workers. Certain types of PPTE are described in the *Diagnostic and Statistical Manual of Mental Disorders* [61] and the Internal Classification of Disease [62] criteria for Acute Stress Disorder and Posttraumatic Stress Disorder diagnosis [63]. Researchers have analyzed how some types of PPTE exposure are associated with mental health disorders [3,6], including recent research results specifically with nurses [14]; however, the current results showcase how some PPTEs involve potentially morally injurious events identified by nurses as impacting their mental health and their patient care responsibilities. Participating nurses reported being unable to assert their care recommendations and that their recommendations are being commonly ignored by physicians in order to adhere to the wishes of patient family members, even when nurse recommendations are aligned with the patient’s wants. Participating nurses described feelings of helplessness and moral distress caused by a lack of professional agency, coupled with undue responsibilities. Nearly all of the ethical and professional dilemmas described by participating nurses involved being unable to provide the best care for their patients.

The current data were collected prior to the COVID-19 pandemic; however, every indication is that nurses working through COVID-19 are now facing more potentially morally injurious events than ever before [9]. Bureaucratic policies and community activities regarding pandemic management have all very likely exacerbated moral challenges related to human or material resource shortages, determining who receives care and in what order, which aligns with the extant difficulties described by participants in the current study as particularly problematic for mental health. There are also indications that COVID-19 has exacerbated the challenges nurses report in the current study related to family members being unreasonable, rude, and confrontational.

Other researchers found in their study of nurses caring for patients with spinal cord injuries that increased attentiveness and sensitivity to nurses’ ethical concerns tended to diminish experiences with, and the magnitude of, moral distress [64]. The *Code of Ethics for Registered Nurses* from the Canadian Nurses Association explicitly mandates nurses to persistently advocate for their patients when they perceive that a medical error has been made or is going to be made [22]; however, cultural, social, and other influences appear to create barriers for nurses to always meaningfully influence patient care in high-stakes situations [65,66]. As Hossain and Clatty note, nurses “affected by this trauma require education, coping tools, and therapy to help avoid or alleviate the adverse effects on their well-being” [53] (p. 23).

In addition, nurses are highly trained in delivering therapeutic communication and, thus, making the necessary structural changes to support their agency may have pervasive benefits for patient care. For example, the Canadian province of Prince Edward Island, in the same way as other jurisdictions across the globe, implemented a Rapid Response Team policy and procedure that, when enacted by a nurse, obligates the presence of two Intensive Care Unit nurses and a respiratory therapist (equipped with a cardiac monitor) to immediately assess declining and critical-status patients. If warranted following their assessment, these healthcare workers will also advocate for the patient to the attending physician. When a Rapid Response Team measure is called, the policy mandates that the assigned physician must physically see their patient within one hour or face professional repercussions. Similar policies in other provinces may provide nurses with an important option for maintaining their own professional standards, supporting patient care, and perhaps mitigating their experiences of moral distress.

Our data imply that all nurses require access to profession-specific mental health supports that can help with the challenging and complex PPTEs experienced by nurses. The same supports may help nurses who are reporting overwhelming experiences with burnout, conflict, and job dissatisfaction [8]. Nurses and patients may also benefit from models of care that allow for coordinated, professionally-respectful, and collaborative team-oriented interventions.

Nurses in our study struggled greatly with the emotional labor, toil, and moral injury that comes with having to console and manage distressed family members—all of which have likely been exacerbated by COVID-19 [9]. If resources are available, issues related to disagreements and conflict between nurses and patient families may be resolved through a multi-disciplinary approach that includes supports from other care professionals, such as social workers or spiritual care workers, who can help share the burdens of care during difficult situations. Family conferences may also be helpful during critical and palliative care situations where all of the directly impacted stakeholders (e.g., the patient, nurses, family members, physicians, social workers) can meet as a team to discuss and clarify prognosis and treatment planning. Family conferences have been effective in working through the difficult emotions, problems, and questions grieving family members encounter when their loved ones are sick, suffering, or dying [67,68].

### Limitations

The current research has several limitations specific to the current study sample and accessibility of the survey itself. The data collection used a large, national, and diverse sample of Canadian nurses; however, the sample for the current study was not proportionally consistent with the distribution of Canadian nurses. The sample that completed the section on PPTE included: (1) A larger proportion of Registered Nurses and smaller proportion of Licensed/Registered Practical Nurses; (2) larger proportions of nurses from Nova Scotia, Saskatchewan, and Alberta; (3) smaller proportions of nurses from Newfoundland, Ontario, and Quebec; (4) a larger proportion of nurses practicing in rural areas relative to the general nursing population (Canadian Institute for Health Information, 2019). The data collection was intentionally designed for anonymous, voluntary, and self-selected participation; as such, there may have been response biases caused by clinical distress or impairment (e.g., nurses with significant symptoms may have self-selected out of participating). Furthermore, the survey did not collect demographics on immigrant status data, and there were insufficient responses about race or ethnicity to analyze. Das Gupta’s intersectional study, which surveyed 593 Ontario Nursing Association members, demonstrated how racism still persists in some healthcare workplaces, and such environments can make it challenging for victims of racism to fight back where there is a presence of fear, lack of managerial support, ineffective institutional responses, and co-worker harassment [69]. Future research should explore how race and ethnicity informs PPTEs and potentially morally injurious events among nurses. Lastly, the current data were collected before COVID-19; as such, subsequent research should explore the impacts of potentially morally injurious events during COVID-19 on the mental health of nurses.

## 6. Conclusions

By analyzing the additional comment sections of the reported PPTE exposure from a large Canadian nursing survey dataset, we were able to highlight an important yet understudied and underrecognized category of PPTE that nurses are experiencing and, thus, requires both scholarly and political attention. Varying forms of PPTE exposure are inherent to the role of nursing; nevertheless, the ethical, moral, and professional dilemmas discussed by our participants represent potentially morally injurious events that could be addressed proactively with specialized training, policy implementation, and better resource allocation [51]. Future areas of study should narrow in on the medical power and relational dynamics at play, considering nursing agency over their occupational responsibility of providing the best patient care while adhering to the wishes and/or work of physicians, co-workers, and patient family members.

## Figures and Tables

**Table 1:** Participant characteristics.

	Participant Responses	Percent
Sample	120	100
Gender		
Male	3	2.5
Female	117	97.5
Age Group		
19–29	18	15
30–39	29	24.2
40–49	15	12.5
50–59	18	15
60+	10	8.3
Unassigned	30	25
Job Length		
Under 1 year	3	2.5
1 to under 5 years	26	21.7
5 to under 10 years	26	21.7
10 to under 20 years	28	23.3
20+ years	37	30.8
Nursing License		
Registered Psychiatric Nurse	3	2.5
Registered Nurse	108	90
Nurse Practitioner	2	1.7
Licensed or Registered Practical Nurse	5	4.2
Other	2	1.7

## Data Availability

The dataset is not publicly available.
